# Incidental Appendiceal Mucinous Neoplasm Found During Appendectomy in a 15-Year-Old Patient: A Case Report

**DOI:** 10.7759/cureus.70350

**Published:** 2024-09-27

**Authors:** Fernando Aguilar-Ruiz, Kevin Joseph Fuentes-Calvo, Sara Fernanda Arechavala-Lopez, Irving Fuentes-Calvo, Luis F Arias-Ruiz

**Affiliations:** 1 General Surgery, Médica Sur, Mexico City, MEX; 2 Surgery, Médica Sur, Mexico City, MEX; 3 Anatomic Pathology, Fundación Clínica Médica Sur, Mexico City, MEX

**Keywords:** adenocarcinoma, appendectomy, appendiceal tumors, mucinous, neoplasm

## Abstract

Appendiceal mucinous neoplasms (AMNs) are rare gastrointestinal tumors, often underdiagnosed due to their variable presentation. Low-grade appendiceal mucinous neoplasms (LAMNs) are particularly significant because of their association with pseudomyxoma peritonei (PMP), a condition that increases the risk of abdominal recurrence. This report presents the case of a 15-year-old female with no prior medical history who developed nonspecific abdominal symptoms. Imaging revealed features consistent with appendicitis, leading to a laparoscopic appendectomy. Histopathological analysis confirmed a low-grade mucinous neoplasm confined to the appendix, with no perforation and clear surgical margins. The case underscores the importance of timely surgical intervention and accurate histopathological evaluation, as early diagnosis and appropriate management are crucial for preventing complications such as pseudomyxoma peritonei. This is particularly relevant in younger patients, where the early onset of such tumors is atypical. The rarity of appendiceal tumors and the need for precise surgical and pathological management are critical to improving patient outcomes and reducing the risk of recurrence.

## Introduction

Appendectomy is one of the most common procedures in general surgery. Following histopathological analysis, approximately 1% of appendectomy specimens will incidentally reveal the presence of an appendiceal tumor [1], a rare condition that accounts for less than 0.5% of all malignant intestinal neoplasms [2]. Adenocarcinoma is the predominant type of primary appendiceal cancer, constituting 60% of cases and showing an incidence rate of 37%-38% among all diagnosed appendiceal cancers. Among adenocarcinomas, the mucinous subtype, or appendiceal mucinous neoplasm (AMN), stands out, representing 8%-10% of appendiceal tumors and 58% of malignant ones [2]. This subtype typically presents around the age of 60, with no gender preference or known risk factors [3]. Often, its course is asymptomatic in 50% of cases [3], although 30% of patients may experience nonspecific symptoms such as abdominal pain or distension [3,4]. AMNs are responsible for most cases of pseudomyxoma peritonei (PMP), a clinical condition characterized by the progressive accumulation of mucinous tumor material in the peritoneal cavity, associated with a poor prognosis and a low survival rate [5].

In 2016, the Peritoneal Surface Oncology Group International (PSOGI) issued a consensus statement on the nomenclature and classification of appendiceal epithelial neoplasms and PMP. According to this document, mucinous neoplasms are classified as follows: low-grade appendiceal mucinous neoplasm (LAMN), which extends into the appendiceal wall without infiltrative invasion and presents low-grade cytological atypia; high-grade appendiceal mucinous neoplasm (HAMN), which also extends into the appendiceal wall without infiltrative invasion but with high-grade cytological atypia; mucinous adenocarcinoma, which shows infiltrative invasion and may be well, moderately, or poorly differentiated; and poorly differentiated mucinous adenocarcinoma with signet ring cell features.

In this context, we present the clinical case of a 15-year-old female patient who presented with an incidental finding of a low-grade appendiceal mucinous neoplasm and discuss its surgical management.

## Case presentation

We present the case of a 15-year-old female patient with no history of personal pathological conditions. During an indirect interview, she reported the onset of loose stools without mucus or blood, followed by a burning-type abdominal pain in the mesogastric region, with an intensity of 5/10. The pain had no apparent trigger and did not radiate. A family member empirically administered 20 mg of omeprazole, but there was no improvement. Subsequently, the pain intensified to an 8/10 and radiated to the right iliac fossa, prompting the patient to take 400 mg of ibuprofen and aluminum hydroxide, which provided partial relief of symptoms. However, due to the persistence of symptoms, she sought emergency care.

**Figure 1:**
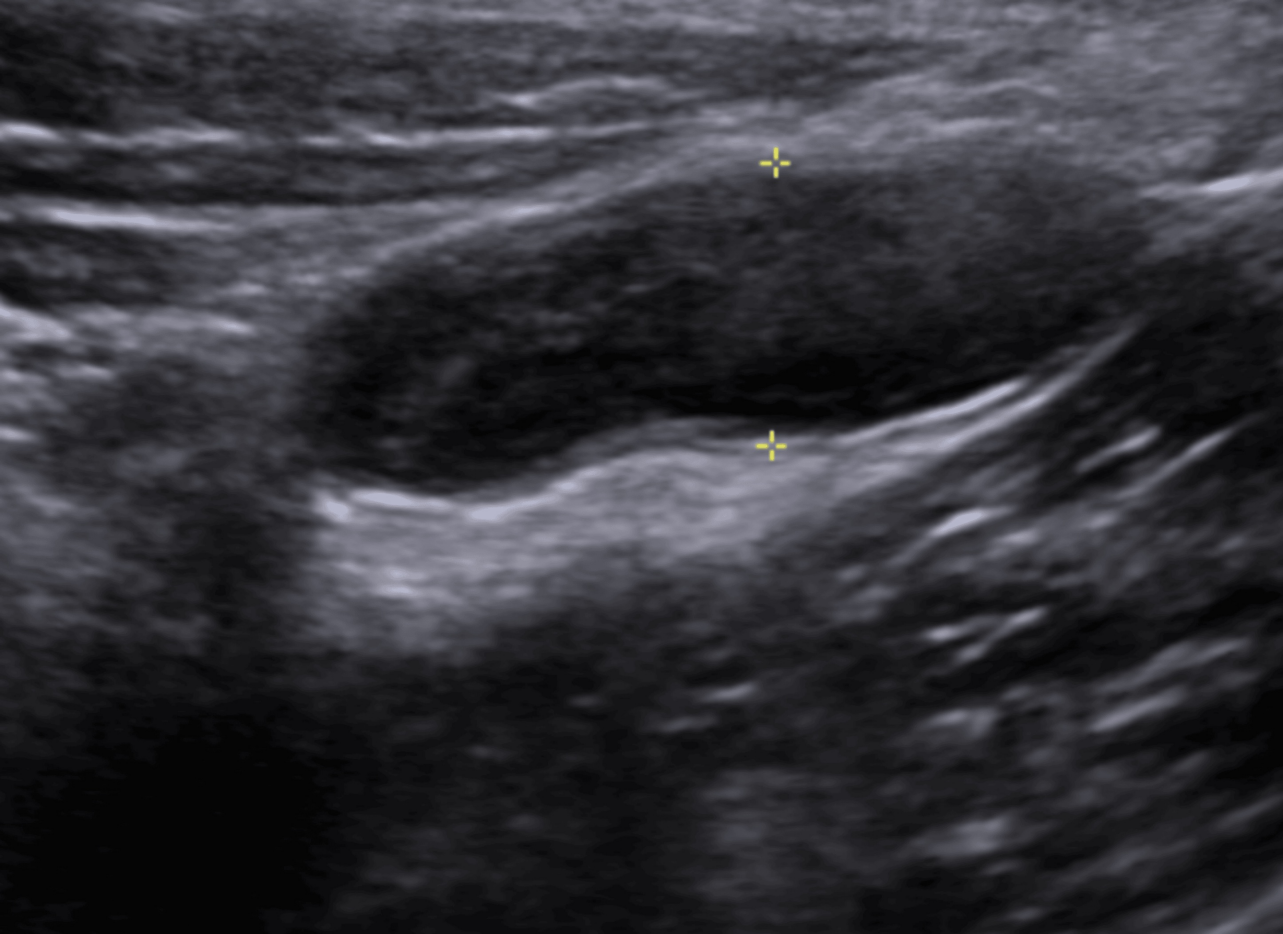
Abdominal ultrasound showing appendicitis Cecal appendix measuring 8.7 mm with free fluid and echogenic pericecal fat

During a focused interview, she denied the presence of fever, vomiting, or loss of appetite. Upon admission, the patient was hemodynamically stable and afebrile, with vital signs within normal parameters. On physical examination, she exhibited positive rebound tenderness in the right iliac fossa and a positive McBurney's point. An abdominal ultrasound was subsequently performed, revealing an 8.7 mm cecal appendix with free fluid and echogenic pericecal fat (Figure 1). Laboratory tests showed no leukocytosis or alterations in inflammatory markers.

**Figure 2:**
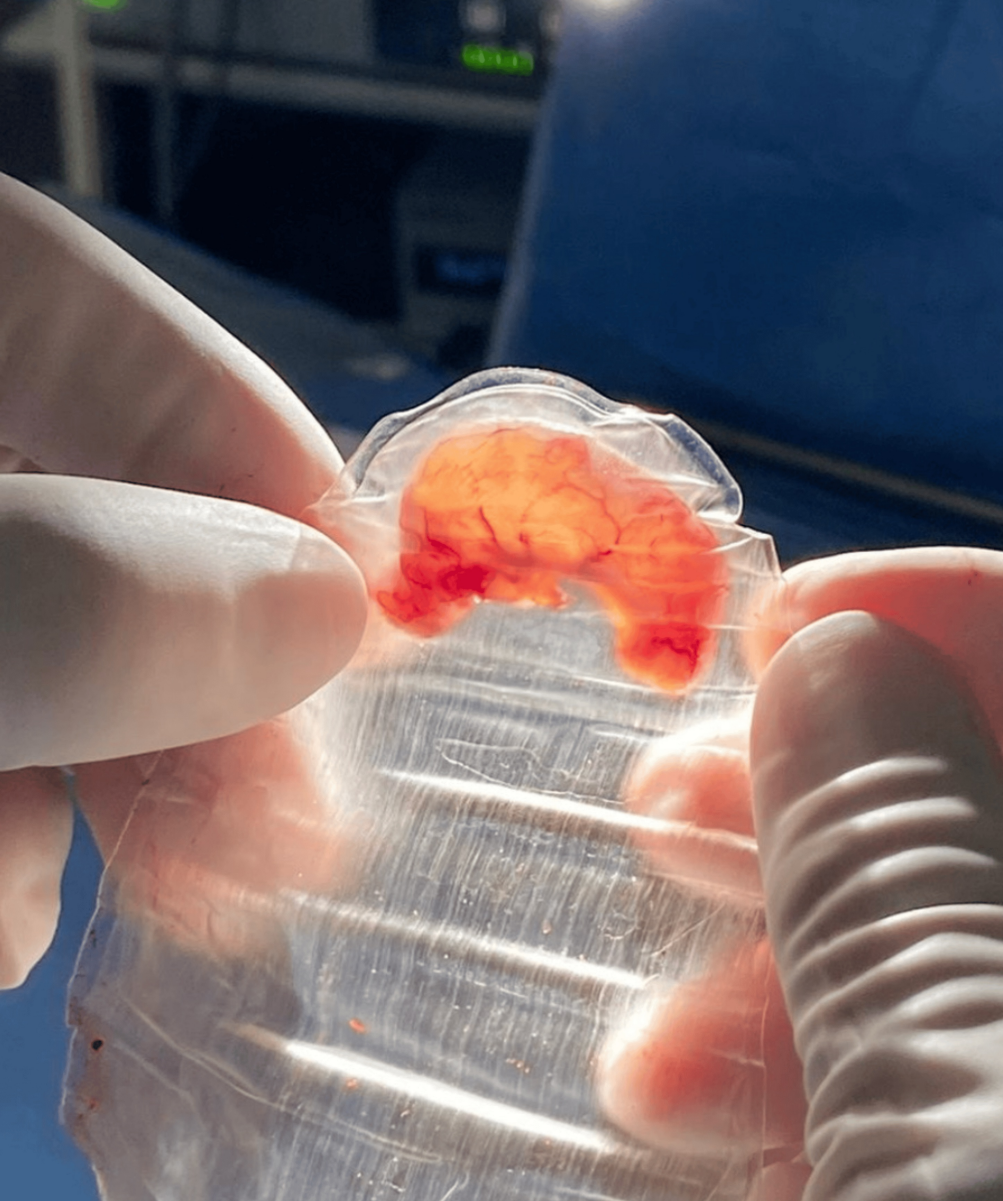
Cecal appendix Appendix measuring 9 × 1.5 cm with mucous content inside and a hyperemic wall

For this reason, a surgical resolution was decided through a laparoscopic appendectomy. A 12 mm transumbilical trocar was placed using the Hasson technique, followed by the placement of two 5 mm trocars in the supraumbilical region and left iliac fossa. The mesoappendix was dissected using ultrasonic energy, and the appendiceal stump was managed with two endoloops. The findings included a 9 × 1.5 cm appendix with mucous content inside, a hyperemic wall, and inflammatory fluid in the pelvic cavity and right iliac fossa. The specimen was sent to the pathology department (Figure 2).

**Figure 3:**
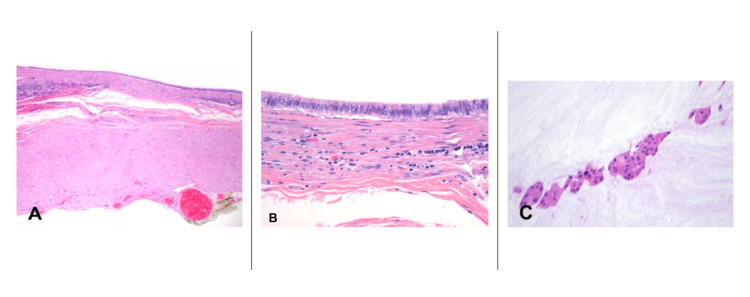
Histology A: Sections stained with hematoxylin and eosin show a thin appendiceal wall with little to no remnant of lymphoid tissue. B: The neoplastic epithelium is simple columnar with mildly atypical nuclei and an abundant eosinophilic mucinous cytoplasm. Notice the few residual lymphocytes. C: The luminal mucin is amorphous and basophilic, and contains floating clusters of neoplastic cells.

The appendix was cystically dilated with mucinous content, with floating groups of neoplastic cells. The neoplastic epithelium was composed of columnar cells, with mild nuclear atypia and abundant mucinous cytoplasm. There were little to no remnants of lymphoid tissue. There were no infiltrative borders, mucinous deposits on the serosal surface, or perforation of the appendiceal wall (Figure 3).

The postoperative course was favorable during the following days of hospitalization, with discharge occurring without complications, no need for reintervention, and follow-up in the outpatient clinic.

In the subsequent days, the pathology department definitively reported a mucinous neoplasm of the appendix with low-grade dysplasia, confined to the appendix without perforation and with clear surgical margins.

## Discussion

Appendiceal neoplasms are among the rarest entities within gastrointestinal tumors [6]. It is important to note that these types of neoplasms are often underdiagnosed, as the condition of the appendix at presentation can vary, and it is sometimes found perforated [7,8]. Additionally, the literature indicates that the incidence is higher in females. However, in this patient, the early onset is particularly notable, given that the average age of presentation is 50-60 years [9]. In all cases of mucinous appendiceal neoplasms, a histopathological examination of the appendix is essential to determine the extent of mucinous epithelium and the depth of acellular mucin deposits. This diagnosis also requires correlation with intraoperative findings, as well as a thorough exploration by the surgeon in the right lower quadrant for any predisposing conditions [10].

Mucinous neoplasms of the appendix can be grouped in a spectrum of mucin-producing tumors, which include mucin-producing adenomas, low-grade and high-grade appendiceal mucinous neoplasms, and mucinous adenocarcinomas [11,12].

Low-grade appendiceal neoplasms (LAMNs), also known as mucinous cystadenoma [12], usually appear during the sixth decade of life and are more common in females. About 15%-20% of all LAMNs are incidental findings during abdominal surgery [11].

This tumor may exhibit nonspecific gross features or may present as dilated, ruptured, or with mucin deposits on the serosal surface. Upon opening the specimen, the mucosa may appear corrugated, granular, or even smooth. The smoothness of the mucosa occurs due to the tension of mucin against the appendiceal wall, leading to cystic dilation of the wall [11].

Histologically, LAMNs show a mucinous neoplastic epithelium with a villous or flat architecture and atrophy of the underlying lymphoid stroma. Epithelial cells feature low-grade cytology, characterized by an atypical nucleus, eccentrically placed due to compression by the abundant cytoplasm filled with mucin. Extracellular mucin pools are seen, which may have floating neoplastic single cells or cell clusters. Mucin within the lumen can accumulate, leading to cystic dilation and flattening of the epithelium [11,12]. Pushing invasion of the appendiceal wall is a common feature of LAMNs, or the mucin may dissect the wall. Eventually, the wall may rupture, and mucin (with or without cells) may deposit onto the peritoneal surface [11,12].

Low-grade appendiceal mucinous neoplasms are associated with the presence of acellular mucin on the surface of the appendix, which is highly significant as it represents a major risk factor for the phenomenon known as pseudomyxoma peritonei, increasing the likelihood of abdominal recurrence [13]. Regarding the surgical procedure, it is crucial to emphasize that the approach depends on intraoperative findings. If there is no evidence of mucin in the abdominal cavity or if the appendix is not perforated, the preferred technique is laparoscopic appendectomy. However, if the appendix is perforated or there is abundant mucin on the visceral surface, a right hemicolectomy or the administration of neoadjuvant chemotherapy is recommended. In both scenarios, it is essential to assess whether the surgical margins of the appendix are free of neoplasia, as it has been demonstrated that clear margins reduce the risk of recurrence within the first three years following surgery. If clear margins are not achieved, the use of adjuvant chemotherapy is recommended.

Another particular case is pseudomyxoma peritonei, where different procedures are recommended, with hyperthermic intraperitoneal and intrapleural chemotherapy (HIPEC) showing the best results [13,14]. This highlights the importance of timely and accurate diagnosis, as well as performing an appendectomy at the appropriate moment. Doing so allows for better preservation of the surgical specimen, enabling an accurate histopathological diagnosis and determining the necessary follow-up for the patient, which is directly related to their prognosis.

Intra-abdominal abscess (IAA) is a known complication after appendectomy. Studies report that the incidence of IAA after laparoscopic appendectomy is approximately 4%-5%, while open appendectomy shows a slightly lower rate of around 2%-3% [15]. Although laparoscopic surgery offers faster recovery and less postoperative pain, it carries a higher risk of IAA. Expectant management, particularly for uncomplicated appendicitis, has been proposed as a nonsurgical option but also presents a risk of abscess formation, which should be carefully considered [16].

In immunosuppressed patients, appendicitis is often difficult to diagnose due to atypical presentations, leading to delayed treatment and increased morbidity and mortality. The decision between immediate appendectomy and expectant management is complex in these patients, as they are more prone to infections and poor wound healing [17].

Additionally, although appendiceal neoplasms are rare, especially in younger patients, they can present at more advanced stages in this group. Literature highlights that advanced-stage neoplasms in younger patients complicate diagnosis and management, requiring a high index of suspicion and potentially more aggressive treatment [18].

## Conclusions

The choice of surgical approach is of paramount importance and should be tailored to the individual patient's condition. Key factors to consider include the structural integrity of the appendix, the presence and distribution of mucin on the visceral surface or within the abdominal cavity, and the extent of tissue involvement at the surgical margins. Depending on these variables, surgical options range from a minimally invasive laparoscopic appendectomy to a more extensive right hemicolectomy. In instances where there is evidence of pseudomyxoma peritonei, a more aggressive treatment approach, such as hyperthermic intraperitoneal chemotherapy (HIPEC), is recommended to manage the disease effectively.
